# Supplementary material for: Lung IL-17A-Producing CD4+ T Cells Correlate with Protection after Intrapulmonary Vaccination with Differentially Adjuvanted Tuberculosis Vaccines
Source: Vaccines (Basel). 2024 Jan 26;12(2):128. doi: 10.3390/vaccines12020128 (PMC10892942; doi:10.3390/vaccines12020128)
Supplement: Supplementary file 1 [file vaccines-12-00128-s001.zip › vaccines-2809781-supplementary.pdf]

Supporting Information

Lung IL-17A-producing $CD4^+$ T cells correlate with protection after intrapulmonary vaccination with differentially adjuvanted tuberculosis vaccines.

Stewart *et. al.*

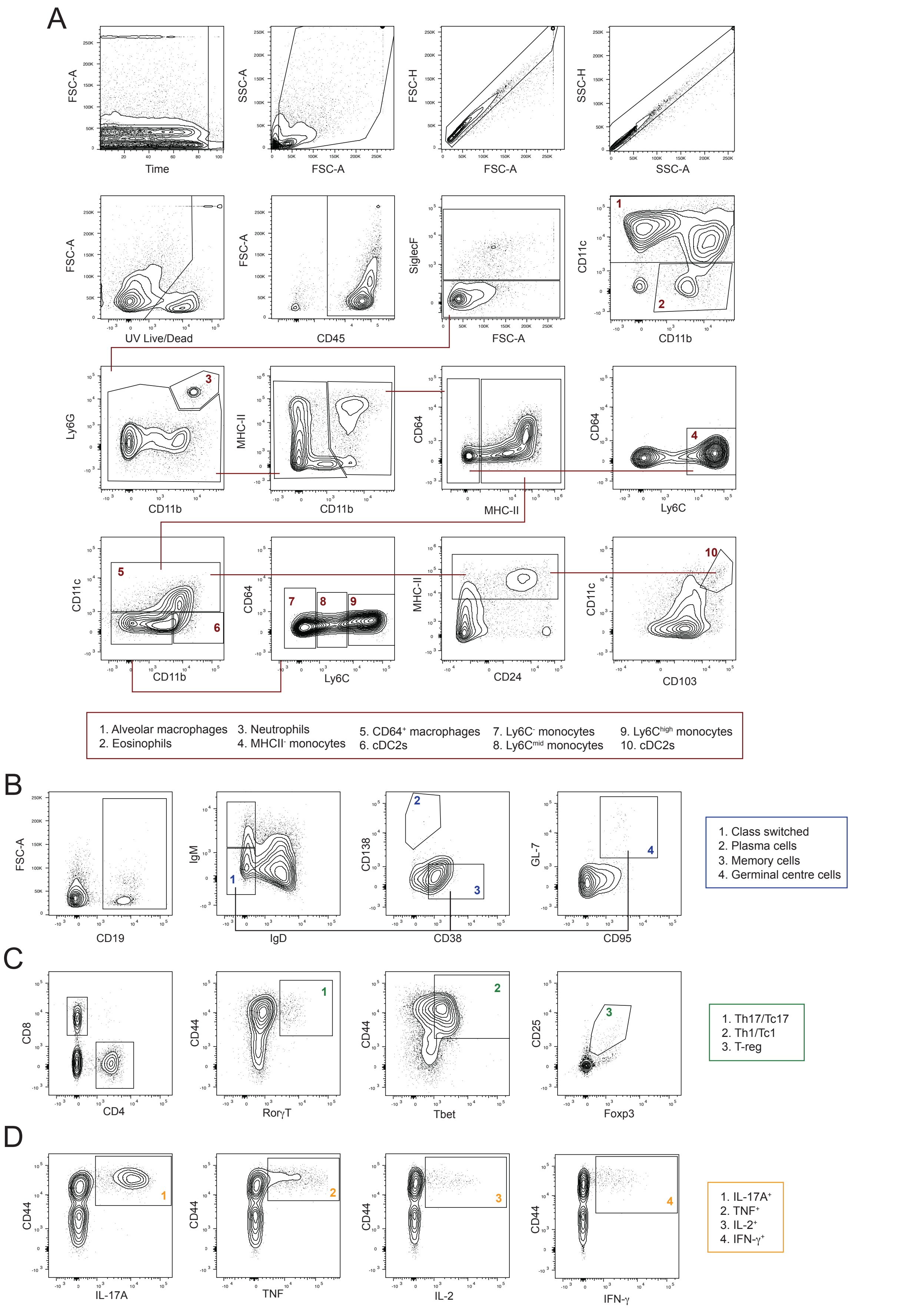

Supplementary Figure S1: Manual gating strategies used in TB vaccine studies.

(A) Cells were first gated on a time gate, debris was excluded followed by the exclusion of doublets, and finally gated on live cells, before myeloid cell subsets were individually identified. (B) Cells were gated as live cells as in (A), then B cells were identified as CD19^{+}, followed by identification of subsets. (C) Cells were gated as live cells as in (A), then T cells were gated on CD4 or CD8 expression. Th/Tc subsets were then identified based on their transcription factor expression. (D) Cells were gated as live cells as shown in (A), then as CD4 or CD8 cells as in (C), and cytokine-expressing populations were identified.

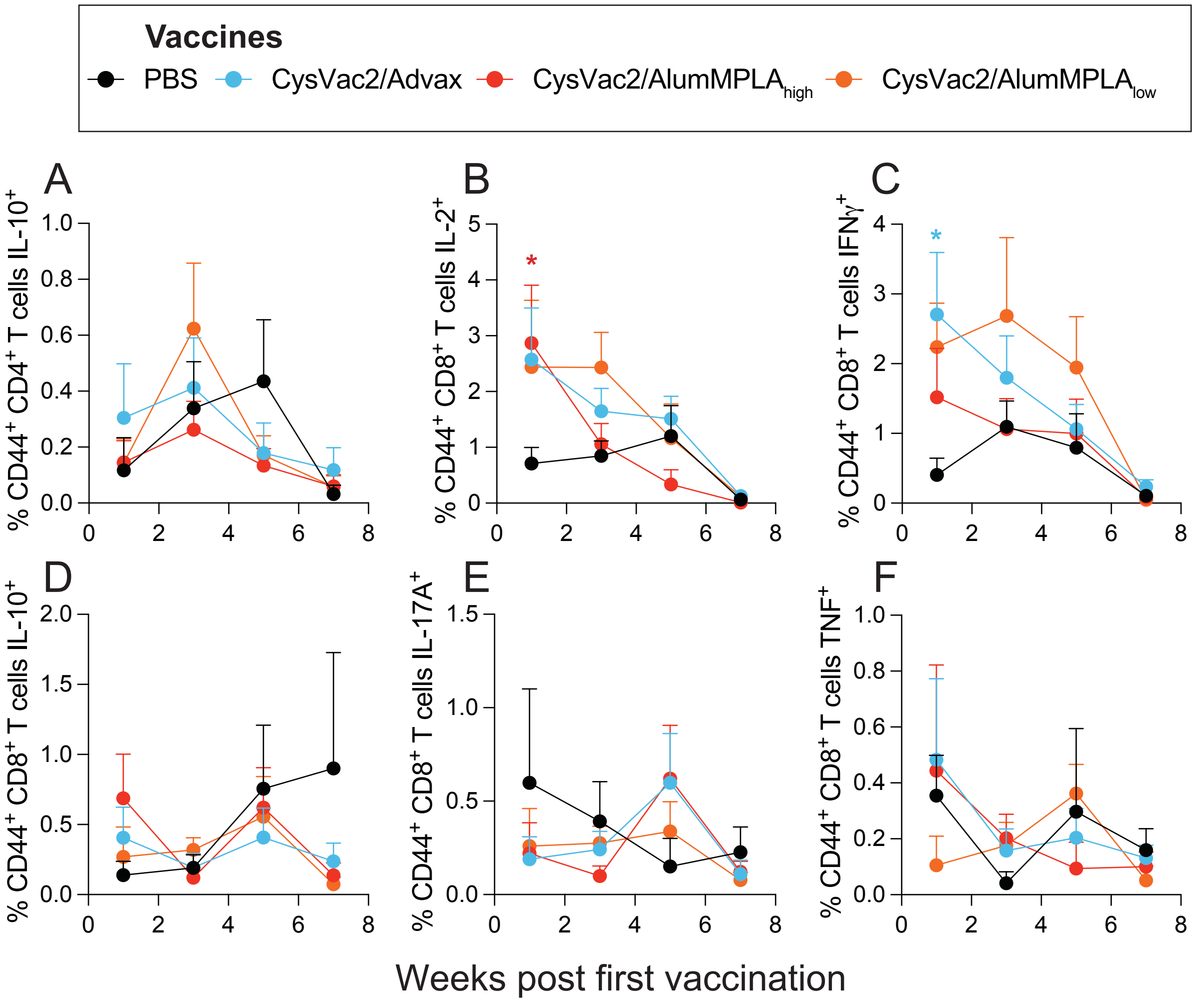

Supplementary Figure S2: Antigen-specific cytokine responses of PBMCs after vacci-nation.

C57BL/6 mice were immunised intratracheally three times two weeks apart, and blood was collected as per the schedule outlined in Figure 1A. After each blood collection, PBMCs were restimulated with CysVac2 protein overnight in the presence of protein transport inhibitor cocktail, and then stained for intracellular cytokine production (A-F). Graphs depict the mean +/- SEM of pooled data from two independent experiments with 5-6 mice per group. Statistics were calculated compared to PBS controls, using a 2-way ANOVA with multiple comparisons, corrected using the Dunnett post-hoc test, $p<0.05$ (*), $p<0.005$ (**), $p<0.0005$ (***).

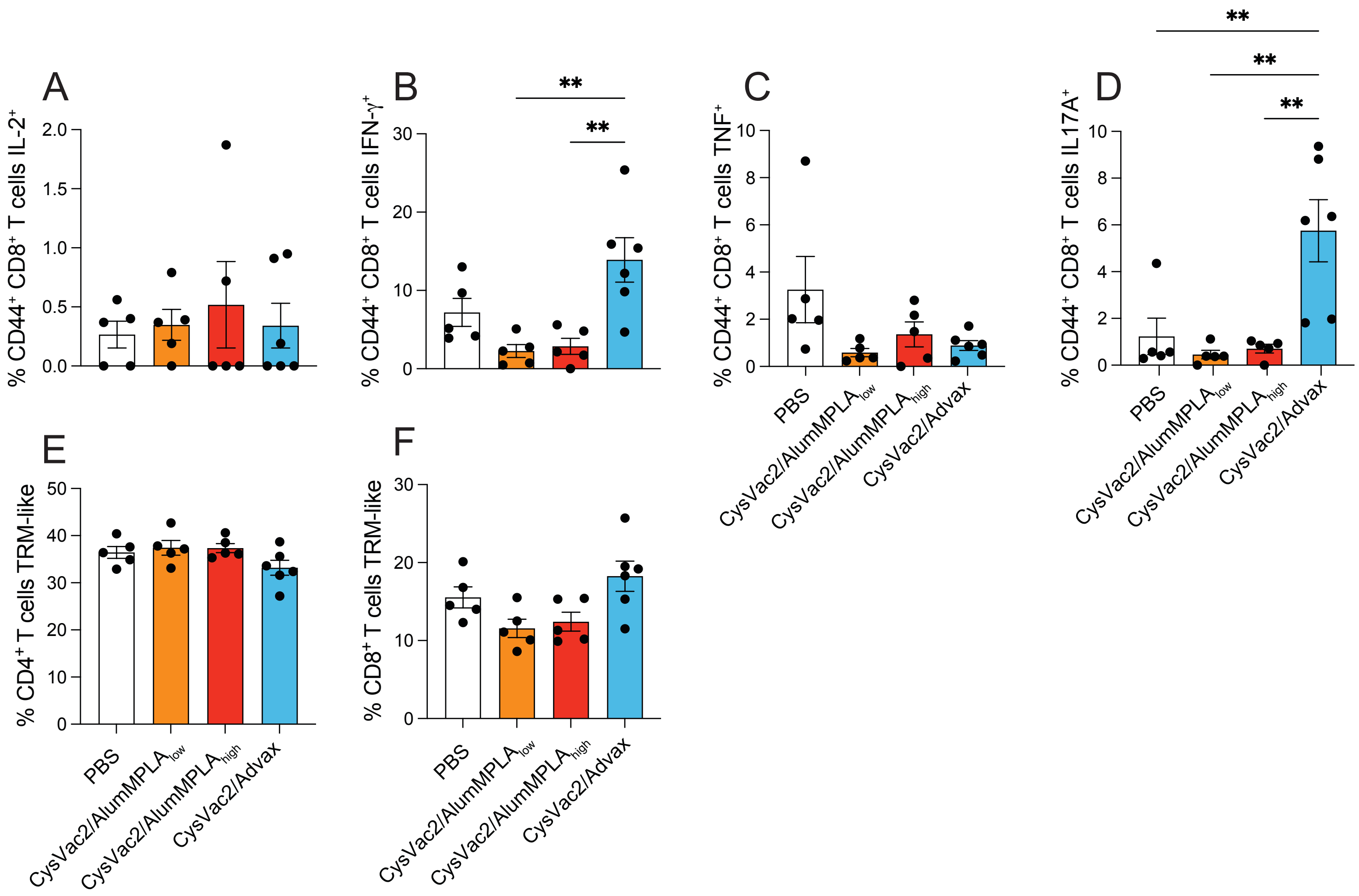

Supplementary Figure S3: T cell phenotypes in the lungs post-infection with *M. tuberculosis.*

C57BL/6 mice were immunised and challenged with *M. tuberculosis* H37rV as described in Figure 1A. 4 weeks after challenge, lungs were collected for flow cytometric analysis. Lung single cell suspensions were restimulated overnight with CysVac2 protein in the presence of protein transport inhibitor cocktail, and then stained intracellularly for cytokine expression. The proportion of lung $CD8^+$ T cells expressing IL-2, IFN-γ, TNF or IL-17A (alone or in combination with other cytokines) is shown in (A-D). $CD4^+$ TRM-like cells (E) were defined as $CD4^+CD44^+CD69^+CD62L^-$, and $CD8^+$ TRM-like cells (F) were defined as $CD4^+CD44^+CD69^+CD62L^-CD103^+$. Graphs are representative of two independent experiments, showing the mean +/- SEM of 5-6 mice per group. Statistics were calculated using a 1-way ANOVA with multiple comparisons, and the Tukey post-hoc test, $p<0.05$ (*), $p<0.005$ (**), $p<0.0005$ (***).

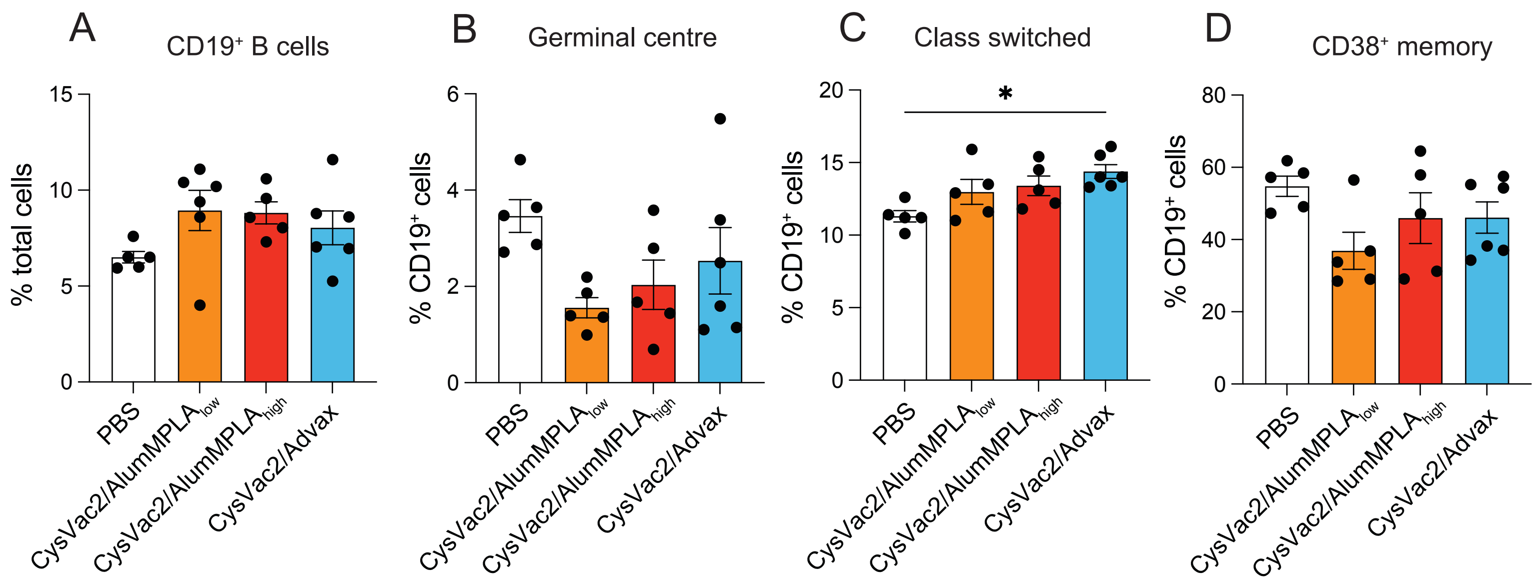

Supplementary Figure S4: B cell phenotypes in the lungs post-infection with *M. tuberculosis.*

C57BL/6 mice were immunised and challenged with *M. tuberculosis* H37rV as described in Figure 1A. 4 weeks after challenge, lungs were collected for flow cytometric analysis. Lung single cell suspensions were stained for B cell phenotypes as defined in the gating strategy in Supplementary Figure 1. Proportion of total lung cells that are $CD19^+$ B cells (A); proportion of lung $CD19^+$ B cells that express CD95 and GL-7 defined as germinal centre B cells (B); proportion of lung $CD19^+$ B cells that are class switched (defined as IgM^- IgD^-) (C); proportion of lung $CD19^+$ B cells that are $CD138^-$ $CD38^+$ memory cells (D). Graphs are representative of two independent experiments, showing the mean +/- SEM of 5-6 mice per group. Statistics were calculated using a 1-way ANOVA with multiple comparisons, and the Tukey post-hoc test, $p<0.05$ (*), $p<0.005$ (**), $p<0.0005$ (***).

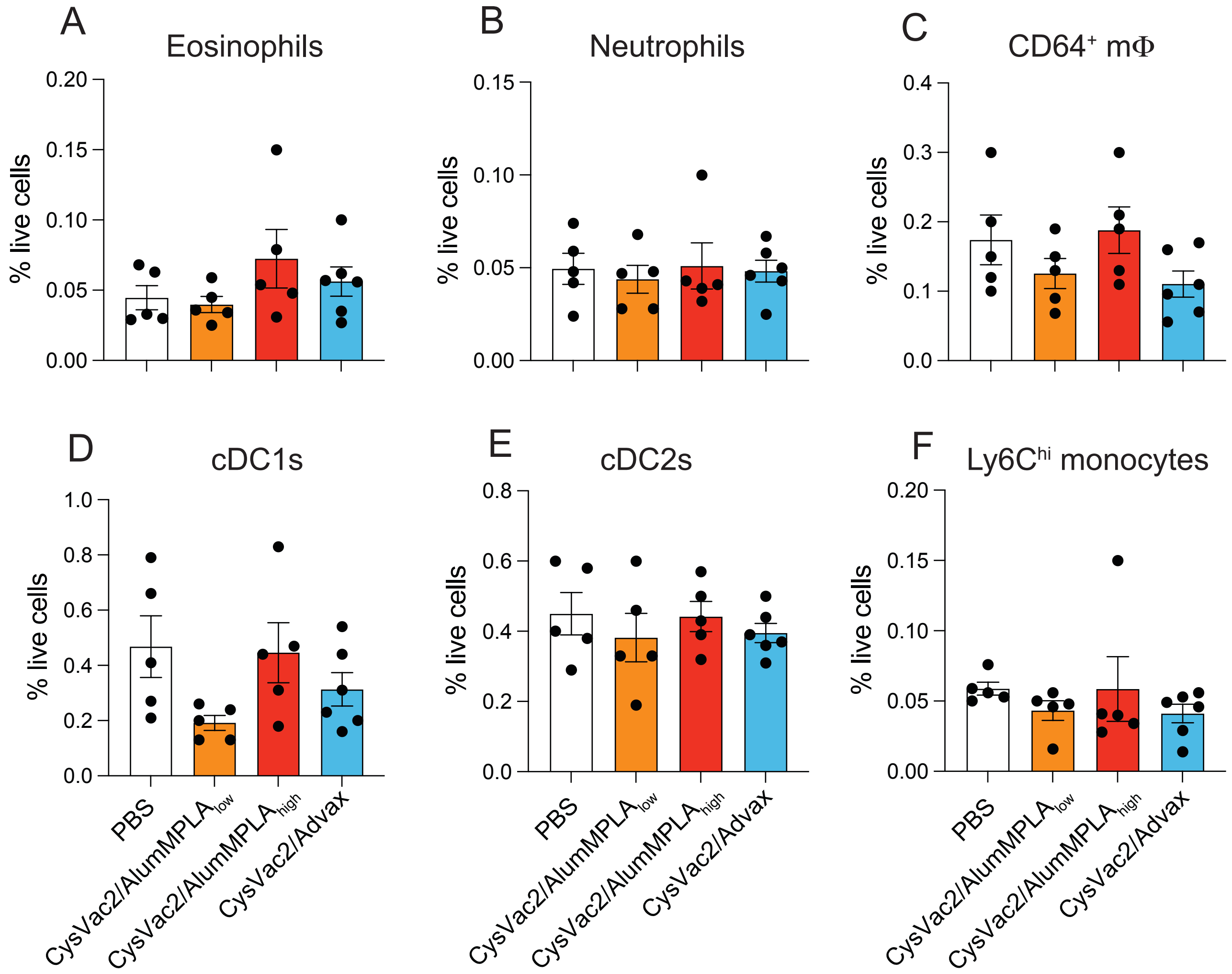

Supplementary Figure S5: Myeloid cell profiles in the mLN post-infection with *M. tuberculosis.*

C57BL/6 mice were immunised and challenged with *M. tuberculosis* H37rV as described in Figure 1A. 4 weeks after challenge, lungs and mediastinal lymph nodes (mLN) were collected for flow cytometric analysis. mLN single cell suspensions were stained for myeloid cells defined in the gating strategy in Supplementary Figure 1. Graphs are representative of two independent experiments, showing the mean +/- SEM of 5-6 mice per group. Statistics were calculated using a 1-way ANOVA with multiple comparisons, and the Tukey post-hoc test, however no groups were significantly different.

Supplementary Table S1: Monoclonal antibodies used for flow cytometry.

Antibody	Clone	Manufacturer
Anti-mouse B220 BUV395	RA3-6B2	BD Biosciences
Anti-mouse CD103 BV711	M290	BD Biosciences
Anti-mouse CD11a BV510	M17/4	BD Biosciences
Anti-mouse CD11b APC/Cy7	M1/70	BD Biosciences
Anti-mouse CD11c AF647	N418	BD Pharmingen
Anti-mouse CD138 BV605	281-2	BioLegend
Anti-mouse CD16/CD32 purified	2.4G2	BD Pharmingen
Anti-mouse CD19 BV786	1D3	BD Biosciences
Anti-mouse CD24 BUV737	M1/69	BD Biosciences
Anti-mouse CD25 PE	PC61	BD Pharmingen
Anti-mouse CD38 APC/Cy7	90	BioLegend
Anti-mouse CD4 AF700	RM4-5	BD Pharmingen
Anti-mouse CD44 BV605	IM7	BD Biosciences
Anti-mouse CD44 FITC	IM7	BD Pharmingen
Anti-mouse CD45 BV510	30-F11	BD Biosciences
Anti-mouse CD62L APC/Cy7	MEL-14	BioLegend
Anti-mouse CD64 PE/Cy7	X54-5/7.1	BioLegend
Anti-mouse CD69 BV786	H1.2F3	BD Biosciences
Anti-mouse CD8 APC/Cy7	53-6.7	BD Pharmingen
Anti-mouse CD8a Pacific Blue	53-6.7	BD Pharmingen
Anti-mouse CD95 PE	Jo2	BD Pharmingen
Anti-mouse Foxp3 PE/Cy7	FJK-16s	eBioscience
Anti-mouse GATA3 BUV395	L50-823	BD Biosciences
Anti-mouse GL-7 AF488	GL7	BioLegend
Anti-mouse IFN-γ PE/Cy7	XMG1.2	BD Pharmingen
Anti-mouse IgD PerCP/Cy5.5	11-26c (11-26)	BD Pharmingen
Anti-mouse IgM BV421	RMM-1	BioLegend
Anti-mouse IL-10 BV605	JES5-16E3	BD Biosciences

Anti-mouse IL-17A BV421	TC11-18H10	BD Biosciences
Anti-mouse IL-2 PE	JES6-5H4	BD Pharmingen
Anti-mouse IL-5 APC	TRFK5	BD Pharmingen
Anti-mouse Ly6C PerCP/Cy5.5	HK1.4	Invitrogen
Anti-mouse Ly6G BUV295	1A8	BD Biosciences
Anti-mouse MHC-II BV421	M5/114.15.2	BD Biosciences
Anti-mouse RorγT PECF594	Q31-378	BD Biosciences
Anti-mouse Siglec-F PE	E50-2440	BD Pharmingen
Anti-mouse T-bet PerCpCy5.5	4B10	BioLegend
Anti-mouse TNF PerCPCy5.5	MP6-XT22	BD Pharmingen
